# Supplementary material for: Protective Effect of Limosilactobacillus fermentum ME-3 against the Increase in Paracellular Permeability Induced by Chemotherapy or Inflammatory Conditions in Caco-2 Cell Models
Source: Int J Mol Sci. 2023 Mar 25;24(7):6225. doi: 10.3390/ijms24076225 (PMC10094186; doi:10.3390/ijms24076225)
Supplement: Supplementary file 1 [file ijms-24-06225-s001.zip › ijms-2262238-supplementary.pdf]

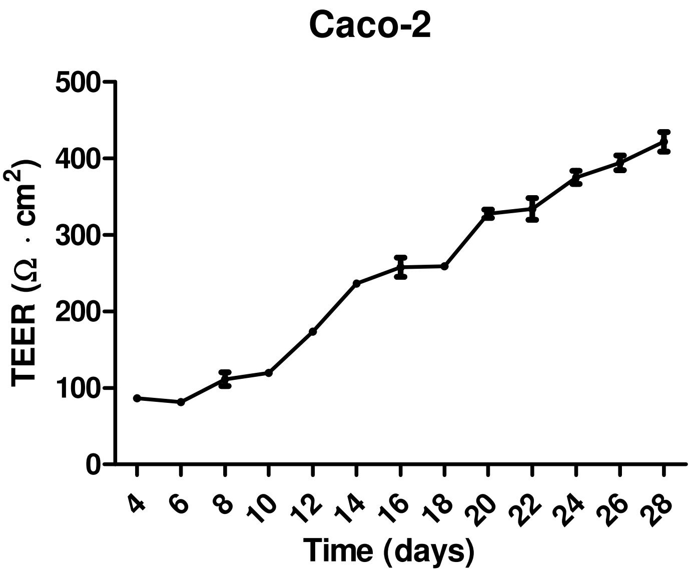

Supplementary Figure S1. TEER value of Caco-2 monolayer cells. Caco-2 cells were seeded on transwell inserts and maintained in culture for 28 days. TEER was measured by epithelial voltohmmeter Millicell®-ERS-2 every two days, before changing the medium, during their differentiation. TEER (Ω x cm^2) was plotted against time. One representative experiment is shown, with mean ± SD from three transwells.
